# Sex and Gender Effects in Recovery From Alcohol Use Disorder

**DOI:** 10.35946/arcr.v40.3.03

**Published:** 2020-11-19

**Authors:** Cathryn Glanton Holzhauer, Michael Cucciare, Elizabeth E. Epstein

**Affiliations:** 1 Department of Psychiatry, University of Massachusetts Medical School, Worcester, Massachusetts; 2 Division of Research and Education, VA Central Western Massachusetts, Leeds, Massachusetts; 3 Department of Psychiatry, University of Arkansas for Medical Sciences, Little Rock, Arkansas; 4 VA South Central Mental Illness Research, Education, and Clinical Center and Center for Mental Healthcare and Outcomes Research, Central Arkansas Veterans Healthcare System, North Little Rock, Arkansas

**Keywords:** sex, gender, treatment, recovery, alcohol, substance use disorder, mechanisms

## Abstract

The current article provides a brief summary of biopsychosocial gender differences in alcohol use disorder (AUD), then reviews existing literature on gender differences in treatment access, retention, outcomes, and longer-term recovery. Among psychotherapies for AUD, there is support for the efficacy of providing female-specific treatment, and for female-only treatment settings but only when female-specific treatment is included. However, despite mandates from the National Institutes of Health to do so, there is little work thus far that directly compares genders on outcomes of specific psychotherapies or pharmacotherapies for AUD. Although existing research has mixed findings on sex and gender differences in overall outcomes, there are more consistent findings suggesting different mechanisms of behavior change among men and women in AUD treatment and long-term recovery. Thus, more work is needed that attends to gender and sex differences, including planning studies that are structured to examine not only gender-differentiated outcomes in treatment response, but equally important, differences in treatment access and attendance as well as differences in mechanisms of change in drinking behavior.

## INTRODUCTION

Between 1994 and 2017, the National Institutes of Health (NIH) issued mandates that biomedical researchers include female participants in clinical research,^1^ analyze sex/gender differences in NIH Phase III clinical trials,^2^ and submit the results from these analyses to Clinicaltrials.gov.^3^ Additionally, between 1992 and 2010, the NIH Office of Research on Women’s Health strategic plan identified sex difference research as a focus in basic science, as well as incorporation of sex difference findings in treatment for girls and women.^4^,^5^ These U.S. national policies and strategic plans have had a profound impact on treatment development for alcohol use disorder (AUD) by accelerating attention to sex and gender differences in research, resulting in increased awareness of gender-specific treatment needs. Currently, evidence-based, female-specific AUD treatments are emerging;^6^ however, there is still insufficient research (or reporting of research results) on gender differences in all areas of research on AUD treatment and its implementation.

Most recent epidemiological results indicate a higher prevalence among men than women of AUD—defined by criteria of the fifth edition of the American Psychiatric Association’s *Diagnostic and Statistical Manual of Mental Disorders* (DSM-5) —with past-year rates of 10% among women and 18% among men, and respective lifetime rates of 23% and 36%.^7^ However, from 2000 to 2013, prevalence rates of 12-month DSM-IV AUD increased by 84% among women compared with 35% among men.^8^ Thus, attention to gender differences in clinical research for AUD is needed, given the steep trajectory of gender convergence over the last 20 years. The current article provides a brief overview of gender differences in biological, psychological, and social aspects of AUD, followed by a review of the existing literature on gender differences in AUD treatment, factors that affect long-term recovery from AUD, and mechanisms of behavior change.

Regarding the terminology used in this article—“sex,” “gender,” and “recovery”—the NIH definition of sex refers to biological differences between females and males in chromosomes, sex organs, and endogenous hormones, whereas gender refers to more socially based roles and behaviors that may vary by historical and cultural contexts.^9^ For this article, American Psychological Association guidelines are used: gender refers to women and men as social groups, and sex refers to the predominantly biological distinction between males and females.^10^

Regarding recovery from AUD, there is currently no consensus in definition of this term. Historically, recovery has been associated with Alcoholics Anonymous as “ongoing cognitive, emotional, behavioral, and spiritual reconstruction of the sobered alcoholic”^11^,^12^ and more recently, “a voluntarily maintained lifestyle characterized by sobriety, personal health, and citizenship.”^13^ In contemporary treatment research, AUD recovery is generally operationalized by primary outcomes related to reduction in drinking, increased abstinence rates, and/or reduction of AUD symptoms. Improvements in secondary outcomes such as other drug use, daily functioning, psychiatric symptoms, physical health, and employment status also are often assessed in AUD clinical trials and are increasingly viewed as outcomes inherent to recovery. Some recent research has focused on the relative importance of abstinence versus reduction of drinking and related symptoms (primary and secondary) in the definition of, and clinical implications for, recovery.^14^ In the current article, the term “treatment outcome” is generally used in lieu of recovery, with the understanding that treatment outcome refers to both primary (drinking) and secondary outcome variables.

Lastly, the research reviewed in this paper uses diagnoses from DSM-IV and DSM-5. Whereas DSM-IV described two distinct disorders—alcohol abuse and alcohol dependence—DSM-5 combines these into a single alcohol use disorder (AUD) with mild, moderate, and severe subclassifications reflecting the number of symptoms met. The main criteria change from DSM-IV is that DSM-5 eliminates alcohol-related legal problems and adds alcohol craving as a criterion for AUD. Lastly, although the search did not exclude international research, the majority of findings reviewed are from studies conducted and/or funded in the United States.

## BIOPSYCHOSOCIAL SEX AND GENDER DIFFERENCES IN ALCOHOL USE AND AUD

### Biological Sex Differences

#### Physical effects of alcohol

Alcohol is consistently shown to have more negative effects on women’s health than men’s, even at weight-adjusted lower levels of alcohol exposure, partly due to gender differences in pharmacokinetics of alcohol.^15^ Because women typically have less total body water and greater total body fat, alcohol is more concentrated in women’s bodies than in the bodies of men, creating greater blood alcohol content at similar doses and weights.^16^ Women with AUD also are more likely to develop alcohol-related heart disease, cancer, and liver disease,^17^ and more overall brain atrophy secondary to chronic drinking.^18^

#### Physiological stress response

Stress plays an important role in the development and maintenance of AUD among both men and women.^19^ Yet, alcohol-induced alterations in emotional and biophysiological markers of adaptive stress response are more common in women than men.^20^ The nature and extent of some alterations are also gender-specific (e.g., blunted physiological responses to stress cues, alcohol cues, and alcohol exposure; sensitized emotional response to stress; alterations in hormonal fluctuations).^21^ Furthermore, inflammatory responses to alcohol exposure, stressors, and trauma are highly sex-specific and have widespread physiological effects.^16^ Such altered responses to stress differentially increase risk for and/or maintain AUD, co-occurring emotional disorders, and/or secondary effects of alcohol use (such as neural degeneration) among men and women.

#### Hormones

Sex hormones affect all body systems directly and indirectly, and for women there appears to be a reciprocal effect of alcohol on sex hormones.^16^ Chronic alcohol use has been shown to affect testosterone levels in men,^17^ whereas female sex hormones (estradiol, progesterone, and their metabolites) reciprocally interact with alcohol use.^16^,^22^ Specifically, alcohol induces alterations in estrogen receptor physiology and function,^16^ which may contribute to osteoporosis, sexual dysfunction, and infertility in women.^17^ Further, sex hormones may influence patterns of women’s alcohol intake.^23^ Research is beginning to elucidate the mechanisms of these interactions. For instance, estrogen levels may enhance the rewarding properties of substances and increase impulsive behavior, whereas progesterone may attenuate substance-rewarding effects.^22^,^23^ Furthermore, decreases in progesterone may increase vulnerability to stress and potentiate stress-induced drinking.^21^

### Psychosocial Gender Differences

#### Co-occurring psychiatric conditions

Women with AUD report higher levels of co-occurring psychiatric conditions than do men with AUD. Co-occurrences of mental health conditions with AUD were examined using data from two waves (2001–2002 and 2004–2005) of the National Epidemiologic Survey on Alcohol and Related Conditions (NESARC).^24^ Women were found to have higher rates of all mood and anxiety disorders as well as paranoid, histrionic, borderline, and avoidant personality disorders compared to men, who had higher rates of narcissistic and antisocial personality disorders. After adjusting for sociodemographic factors, among persons reporting alcohol abuse (not dependence), only major depressive disorder was identified to be more likely among women than men. Recent research by Karpyak et al. found that women with AUD, compared to men with AUD, had higher rates of lifetime major depression, substance-induced depression, anxiety disorder, and post-traumatic stress disorder (PTSD) and were more likely to drink alcohol when experiencing negative emotion.^25^ Further, among U.S. military veterans with AUD, women report more co-occurring mental health and substance use disorders than do men.^26^

#### Mood and coping factors

Among individuals with AUD, women are more likely than men to experience alcohol cravings in response to daily negative emotion and stress.^20^,^21^,^25^ In a sample of adults with PTSD and AUD, drinking to enhance positive emotions was associated with alcohol use in both men and women, whereas drinking to cope with negative affect was associated with higher alcohol consumption in women but not men.^27^ Another study reported a positive association of negative affect with alcohol cravings for men at the beginning of alcohol detoxification, but for women the association persisted throughout detoxification.^28^ Additionally, for women, more depressive symptoms at the beginning of detoxification were associated with more alcohol cravings at the end of detoxification. A third study also found that women were more likely to report high anxiety and depression at alcohol detoxification admission and discharge compared to men.^29^ In that study, both genders showed increased anxiety and depression symptoms at 6-month follow-up, with more anxiety symptoms predicting men’s relapse at 12-month follow-up and more depression symptoms predicting women’s relapse at 12-month follow-up.^29^

#### Trauma exposure

There are high rates of trauma among women receiving treatment for any substance use, and an estimated 25% to 55% of women in substance use treatment have PTSD.^30^ Trauma and acute stressors are causally associated with the development of AUD in women, via the effects of stress and trauma on biological processes and the likelihood of women with AUD to drink to cope with negative emotion and stress.^20^ One study examining childhood maltreatment and lifetime odds of AUD found that, for both genders, having a history of physical, sexual, and/or emotional abuse and/or physical and/or emotional neglect was associated with higher odds of having a lifetime AUD.^31^ For women, the strength of the relationship between lifetime AUD and all types of childhood maltreatment, except emotional abuse, was stronger than for men. In addition, Heffner and colleagues found that, for women, severity of current trauma symptoms and number of lifetime traumas predicted relapse over the course of the study.^32^ No association between trauma and relapse was found for men.

#### Social networks

Research has found gender differences in the relationship between social networks, social support, and alcohol use. For example, compared to men, women with AUD are more likely to have a family history of AUD and a spouse with a history of AUD.^33^ Women also are less likely than men to have social support in their recovery.^15^ This may be at least partly due to greater stigma related to women’s alcohol use compared to men, or to women’s fear of interpersonal consequences related to their drinking.^34^ Indeed, women tend to be more isolated in their excessive alcohol use and recovery.^15^ Men report greater social pressure to change their drinking behaviors than women.^35^ However, a study using data from the National Alcohol Study between 1984 and 2010,^36^ with data from more than 32,000 people, showed changes over time for women. Although results did show that men displayed overall greater incidences of pressure to change across the years, there was also a significant cohort effect for women, with younger cohorts of women (i.e., born after 1964) reporting greater social pressure to change drinking. Such results coincide with gender convergence in rates of AUD and suggest that there also may be an emerging convergence of social pressure to change drinking. The role of social networks in drinking is evident in predicting treatment outcomes, reviewed below, and is an important risk and maintenance factor for AUD in men and women—albeit in different ways.

### Summary

Research has illuminated gender differences in the biopsychosocial factors contributing to the development of, and recovery from, AUD. The physical effects of alcohol are more pervasive for women than men, and sex-specific factors, such as sex hormones, have been associated with alcohol use. In terms of psychosocial differences, stress, trauma, and negative affect are particularly relevant contributors to alcohol use and development of AUD among women. Relatedly, there are gender differences in terms of rates of co-occurring mental health conditions, the rates of major depressive disorder among women with alcohol abuse being particularly high. These differences provide a context for understanding potential gender differences in AUD treatment and recovery and can be used to guide future research.

## GENDER DIFFERENCES IN TREATMENT ENTRY, RETENTION, AND OUTCOME

### Treatment Entry

A small percentage of individuals with AUD ever receive treatment, with past-year estimates of 7% of men and 5% of women with AUD receiving treatment^37^ and lifetime estimates of 22% to 23% for men and 15% for women.^38^,^39^ There are several female-specific barriers to accessing AUD treatment, such as external and internalized stigma, lack of childcare, and systemic barriers.^6^ Women are more likely than men to believe their alcohol problem will resolve on its own.^6^ Additionally, women who are of minority racial or ethnic groups, of different sexual orientations, in the criminal justice system, living in rural areas, and/or of older age and women who speak languages other than English represent intersectional identities that add barriers to treatment entry.^40^

Among individuals who do enter AUD treatment, there are gender differences in clinical presentation. Women tend to have more severe alcohol and drug use histories, lower education and income, higher unemployment and housing needs, more children living at home, and higher parental stress, and they tend to be younger in age.^15^ Primary care settings are a useful portal for AUD treatment access, and for women even more so.^41^ Research consistently has found that women access AUD treatment via portals other than specialty AUD options, tending to receive AUD care in mental health and primary care settings.^6^,^15^,^16^,^42^–^44^

### Treatment Retention

Data on gender differences in treatment retention are mixed, and most studies have been completed among samples with substance use disorder (SUD), meaning the results are not specific to AUD. For example, a review by Greenfield and colleagues reported no overall gender differences in SUD treatment retention but hypothesized that there would be different predictors and mediators of retention among men and women.^42^ Among both genders, treatment retention has been associated with higher financial resources, fewer mental health problems, less severe substance use problems, more employment, and older age. Female-specific factors related to SUD treatment retention include referral source, personal stability, number of children, and availability of childcare.^42^ A separate study found that type of care setting (i.e., detoxification, residential, ambulatory) also may moderate care retention, with women more likely than men to leave a detox facility prematurely.^45^

### Treatment Outcome

The following review on outcomes of psychosocial treatments for AUD focuses on empirically supported treatments identified by American Psychological Association Division 12.^46^ The pharmacotherapy section focuses on medications approved by the U.S. Food and Drug Administration for treatment of AUD. Search terms included the treatment name (e.g., “motivational interviewing” or “naltrexone”) + “gender” or “sex” + “alcohol.” The authors also searched ClinicalTrials.gov for clinical trials on these AUD treatments, and reviewed publications from large clinical trials for AUD, to determine whether gender differences were analyzed and reported. Lastly, the authors searched for and reviewed reports of clinical trials, literature reviews, or meta-analyses on specific treatments to identify commentary or results regarding sex or gender. This was done to address the fact that analyses not yielding any significant gender differences may not have been identified using the search terms. Thus, for some treatments the authors were able to comment on null gender difference findings. Despite the NIH mandate to include females in biomedical research,^1^,^2^ relatively few AUD treatment outcome studies have reported on gender as a moderator of treatment outcome. The more recent NIH policy mandating analysis and reporting of gender differences in treatment outcomes^3^ should result in deepened knowledge of gender differences in response to treatment and in gender-specific mechanisms that help explain treatment effects.

### Psychotherapy

#### Motivational enhancement therapy, cognitive behavioral therapy for AUD, and twelve-step facilitation

Motivational enhancement therapy (MET) is a psychotherapy that helps patients resolve their ambivalence about engaging in treatment and reducing or stopping their substance use. Cognitive behavioral therapy (CBT) is an approach that focuses on the reciprocal effects of cognitions, emotions, and behaviors that maintain problem drinking. In treating SUD, CBT also focuses on identifying and resolving factors that reinforce or punish the substance use behavior and teaching both general coping skills and coping skills to negotiate drinking triggers. Twelve-step facilitation (TSF) treatment for AUD is based on the traditional Alcoholics Anonymous (AA) 12-step model and focuses on AA attendance, personalized spirituality, and guided introspection (“step work”).

MET and CBT are among the most widely researched treatments for AUD;^47^ however, there has been limited research examining gender differences in the effects of these treatments. Project MATCH (Matching Alcoholism Treatment to Client Heterogeneity) generated studies on gender differences in treatment efficacy, although the samples of the three conditions (CBT, MET, and TSF) were between 70% and 80% male.^48^ Project MATCH had a gender matching hypothesis, positing that women receiving CBT would have better outcomes than women in the TSF condition, a difference that would be greater among women than men. This hypothesis was based on the expectation that CBT would better address secondary issues (such as mood and stress) and that TSF could exacerbate stigma and guilt among women.^49^ This hypothesis was not supported, with women in the TSF aftercare arm attending more AA meetings and reporting more AA involvement than men. CBT was ultimately not found to improve secondary issues to a greater extent than TSF.^49^

Witkiewitz, Hartzler, and Donovan tested whether matching patients’ motivation level to CBT or MET was associated with better outcomes in the aftercare arm of Project MATCH.^50^ Men with lower baseline motivation and above-average alcohol dependence severity were found to drink more frequently in the MET than in CBT condition; the authors proposed that this more severe group may not have done as well in the lower-intensity MET treatment. Women with low motivation (regardless of severity, but who had overall fewer AUD symptoms than men), as well as low-motivated men with below-average AUD severity, reported less frequent drinking in MET compared to CBT. Another study on the outpatient arm of Project MATCH found that, compared with women, men showed greater increases in abstinence self-efficacy over time and across all treatment conditions.^51^

A meta-analysis on controlled trials of brief motivational interventions examined gender as a moderator of treatment effect.^52^ The study was able to generate aggregate effect sizes only for two studies, which did not show evidence of differential response between genders. In a meta-analysis of 22 studies on motivational interviewing, only one study reported on gender effects, with no differences between men and women observed on treatment outcomes.^53^ A meta-analysis of 53 randomized controlled trials (RCTs) testing CBT for SUD found that the percentage of female participants in each study was positively associated with effect size, suggesting that women may benefit more from CBT than men, but these results must be interpreted with caution, as women comprised only 29% of the total sample.^54^

#### Alcohol behavioral couples therapy

Couples-based approaches to the treatment of AUD are based in the assumptions that partners engage in malleable behaviors that reinforce and/or punish the client’s drinking behaviors, and that enhancing intimate relationships can improve problem-solving, enhance relationship functioning, and reduce likelihood of relapse. Behavioral couples therapy (BCT) and Alcohol BCT (ABCT) have been shown to be effective at increasing rates of abstinence from alcohol, decreasing alcohol-related problems, and improving relationship functioning.^55^,^56^ Only one study to date has directly compared BCT outcomes by gender: O’Farrell et al. compared treatment outcomes among men and women with AUD and their partners receiving BCT in a naturalistic setting (not a clinical trial).^57^ Results revealed few differences between genders, with large treatment effects in drinking reduction and small to medium effects in improved relationship satisfaction across the entire sample.

Several studies have tested ABCT separately among samples of men and women. An early study among men with alcohol dependence and their female partners compared three conditions: (1) ABCT, in which the spouse attended all sessions that included both alcohol- and marital-focused treatment; (2) full spousal attendance but alcohol-focused treatment only; and (3) minimal spousal involvement in alcohol-focused individual treatment.^58^ Participants in the ABCT condition showed greater drinking reductions and improvements in relationship functioning compared to those in the other conditions. A second study randomized men with AUD and their partners to either ABCT, ABCT and relapse prevention, or ABCT and AA facilitation; this study found no differences in outcome across treatment conditions but high rates of abstinence across all three conditions.^59^

ABCT also has been tested among women with AUD, and one study compared ABCT to a treatment arm in which women received individual CBT for AUD.^60^ In that study, however, 31% of the women refused the couples’ study arm due to the need to bring their male partner.^61^ The women who did participate in ABCT had slightly more days abstinent and fewer heavy-drinking days at follow-up than did women in the individual CBT arm. In response to women’s preference for individual treatment—yet recognizing the positive results of ABCT and the role significant others play in women’s drinking—a separate study compared ABCT to a “blended-ABCT,” in which women with AUD attended five sessions individually and seven with their male partner.^62^ Results showed equal outcomes across conditions. Thus, ABCT yielded excellent outcomes for men and women with AUD in separate studies, but gender differences in the effects of, and engagement in, ABCT have yet to be directly tested.

### Pharmacotherapy

Three medications are currently approved by the U.S. Food and Drug Administration for the treatment of AUD: acamprosate, naltrexone, and disulfiram. There are important gender differences in their bioavailability, distribution, metabolism, elimination,^63^ and side effects,^64^ highlighting the importance of examining sex as a moderator of medication treatment efficacy for AUD.

#### Acamprosate

A meta-analytic study examined acamprosate for AUD treatment separately for men and women from a total of 22 studies,^65^ some of which included women and some of which did not. Patient data were accessed from 1,217 women and 4,794 men across the studies. Results showed no gender differences in any measure of acamprosate efficacy, safety, or tolerability (including percentage of abstinent days, heavy drinking, study completion, and medication compliance). Another study examined gender differences in treatment outcomes of the Combined Pharmacotherapies and Behavioral Interventions (COMBINE) study.^66^,^67^ Participants in COMBINE received medication management with 16 weeks of placebo, naltrexone, acamprosate, or their combinations, with or without a combined behavioral intervention (a combination of empirically supported interventions from different therapies). Analyses showed that acamprosate was no more effective than placebo when separately analyzed in both men and women.

#### Naltrexone

One of the first studies on naltrexone for AUD was a multicenter, placebo-controlled RCT of injectable naltrexone,^68^ with each condition comprising 32% women. Results showed that naltrexone was efficacious for men, but not women, in terms of reducing heavy drinking. Another study tested outcomes of psychotherapy with either oral naltrexone or placebo and found that naltrexone was not efficacious compared to placebo for female participants in reducing drinking, but it did delay the onset of drinking after an initial lapse.^69^

A third study tested high-dose naltrexone in men and women with co-occurring cocaine use disorder and AUD in a double-blind placebo RCT.^70^ Participants were randomized to receive either naltrexone (150 mg) or placebo (58 men and 24 women in each condition), combined with either CBT or medication management. Women taking naltrexone used more cocaine and alcohol than did men and the placebo group, whereas men in the naltrexone group used less cocaine and alcohol compared to women and the male placebo group. The authors hypothesized that side effects of naltrexone (e.g., nausea, vomiting) for women may account for this effect. Indeed, women have been shown to have more negative side effects from naltrexone than men, which may be related to women’s greater sensitivity to the endogenous opioid system.^71^ Women’s sensitivity to the effects of naltrexone also may vary across the menstrual cycle, with greater sensitivity in the luteal phase (i.e., post-ovulatory, late phase of the cycle) compared to the early follicular phase (i.e., preovulatory, early phase of the cycle).^72^

Thus, early studies suggested naltrexone for AUD was not as effective for women as for men, or that women may experience worse side effects, contributing to worse outcomes. However, more recent research has suggested that these effects may be due to study characteristics such as sample size or outcomes assessed. Baros, Latham, and Anton used data from two RCTs comparing a naltrexone plus CBT group and a placebo plus CBT group and found effect sizes favoring naltrexone in men compared to women on some outcomes (drinks per drinking day), but not others (percentage of days abstinent, percentage of heavy drinking days).^73^ A review of naltrexone RCTs among women suggested that the medication may have modest effects for women in drinking quantity and time to relapse, but not on drinking frequency.^74^ However, the number of studies reviewed was small, and additional research is needed.

A secondary analysis of COMBINE data tested treatment effects separately in men and women and found that both genders had better treatment response when they received naltrexone with either medication management or combined behavioral intervention (a combination of empirically supported interventions), in comparison to placebo and any other combination of treatments.^66^ The authors concluded that naltrexone is effective among women, and that studies showing noneffectiveness among women may be due to inadequate sample sizes.

#### Disulfiram

In 2016, Agabio et al. cited the low number of women in clinical trials on disulfiram that preclude evaluation of sex differences in efficacy and safety.^75^ A search for any additional trials since 2016 (search terms “sex” or “gender” or “women” + “disulfiram”) did not yield new information on sex differences in the effect of disulfiram for alcohol use.

### Digital and Mobile Treatment Technologies

Emerging digital and mobile models of treatment delivery include platforms such as telehealth sessions via videoconference; direct access computer programs such as CBT4CBT;^76^ smartphone applications (apps) such as the Addiction—Comprehensive Health Enhancement Support System (A-CHESS)^77^ to help patients track their drinking and provide real-time assistance with coping skills; and therapist text-messaging protocols.^78^

The preliminary research on access and use of AUD treatment via digital and mobile technologies suggests gender differences. For instance, a survey of members of an online social network site for women trying to resolve alcohol problems revealed that 47% of the site’s members had never tried any other form of support related to their drinking.^79^ A large survey study in the United Kingdom showed that women were more likely than men to use online recovery groups (but not recovery websites or apps).^80^ A separate study examining use of one social network site for SUD recovery also found a higher proportion of women than men using the site.^81^ Secondary analyses of an effectiveness trial testing a computer-assisted behavioral intervention (compared to treatment as usual) did not find gender to moderate the effect of treatment condition; however, results did show that acceptability of the computerized intervention was positively associated with abstinence among women, but not men.^82^ Digital and mobile treatment technology for AUD is a burgeoning area of research, which should include analysis and reporting of gender differences in both access and outcomes going forward.

### Summary

Existing research suggests no major gender differences in terms of overall outcome in psychosocial or pharmacological treatments for AUD. However, this finding is qualified by the small number of studies that directly test gender differences and the low enrollment of women in clinical trials. Additionally, as demonstrated by secondary analysis of Project MATCH, moderating factors such as AUD severity and motivation may be differentially associated with outcomes for men and women.

## SEX AND GENDER DIFFERENCES IN LONG-TERM RECOVERY

### Gender Differences and the Broader System of Recovery Care

Recovery is a complicated construct, ill-defined and historically confined to a mutual care, 12-step “disease model” system that considers abstinence as the only viable outcome.^12^ AUD is now conceptualized as a chronic, relapsing medical condition and is thought to require a continuum of care, ranging from acute stabilization to ongoing, post-treatment monitoring and maintenance of recovery, and in need of clear benchmarks of disease resolution.^12^ In this complicated context, gender differences in recovery historically have been understudied, but there are some limited findings, for instance, on AA use and clinical outcomes. As more sophisticated treatment approaches and definitions of target outcomes (including “recovery”) are developed in the field, there will be an accelerated need to identify moderating variables (including gender and other demographic variables) that predict treatment outcomes. The following sections highlight aspects of the intersection between gender differences and recovery research.

### Gender Differences and Mutual Help Groups

Alcoholics Anonymous, the largest and most popular mutual help organization available, offers primarily mixed-gender meetings, but also some single-gender meeting options (i.e., men-only, women-only). However, AA meeting content is consistent across groups and does not necessarily include gender-specific content.^83^ One gender-specific and secular mutual help organization is Women for Sobriety, which provides coping skills and reciprocal support for participants.

Outcomes of single-gender versus mixed-gender AA meeting attendance have not been studied; however, studies on gender differences in treatment outcomes among attendees of mixed-gender AA have shown some significant results, including different moderators of attendance for men and women. One longitudinal study followed 466 men and women for 16 years who were initially untreated for problem drinking.^84^ Women were more likely to participate in AA, had longer stays in inpatient treatment for alcohol in the year after baseline, achieved better outcomes than men at 1 and 8 years, and benefited more from AA attendance during years 2 through 8. At 16 years post-baseline, women were more likely than men to participate in treatment and in AA, to be free of drinking problems, to consume less alcohol, to have fewer DSM-IV dependence symptoms, and to report less drinking to cope and higher abstinence self-efficacy;^85^ women were also more likely to report improvements in depression, friendships, problem-solving, self-confidence, and family relationships and social functioning, compared to men.

Witbrodt and Delucchi followed participation in AA for 7 years and found that men were more likely to stop attending over the 7-year period.^86^ Women with higher co-occurring drug severity were less likely to participate in AA than were women with lower drug severity. Women with more severe psychiatric symptoms were more likely to attend AA than women whose symptoms were less severe. Lastly, men who were less religious and those with networks supportive of drinking were less likely to attend AA treatment. Another study that followed 96 women and 180 men for up to 3 years found that AA membership increased participants’ odds of achieving a year of abstinence, an association that was stronger for women than for men.^87^ Comparing men and women in the United States and Sweden, the odds of AA attendance was greater for women who were both alcohol and drug dependent (versus just alcohol), and for women, the odds of AA attendance increased with the number of friends with whom to talk about personal problems.^83^

In sum, research on gender differences in outcomes of AA attendance are mixed, but the most consistent findings suggest women are more likely to stay in AA longer than men, and there may be different moderators (e.g., drug use, psychiatric comorbidity, religiosity, social networks) of the efficacy of AA for men and women.

### Gender Differences in Response to Continuing Care Interventions

In line with contemporary notions of AUD and SUD as chronic, relapsing diseases requiring a continuum of care, McKay and colleagues developed and tested stepped and continuing care interventions with various levels of intervention, including telephone counseling.^88^,^89^ The continuing care approach has implications for women with AUD, for whom social networks supporting abstinence may be particularly relevant for maintenance of recovery.

In a sample of participants who used cocaine, most of whom were also alcohol dependent, McKay and colleagues found that women but not men benefited from telephone continuing care.^89^ Further study of gender moderators revealed lower rates of cocaine-positive urine for women at 24 months, but not men, if receiving telephone continuing care versus treatment as usual.^90^ More work is encouraged in this area for AUD; sample sizes of women need to be sufficiently large to test for gender differences, and social support for abstinence and emotional support should be incorporated.

### Precipitants to Relapse

Sliedrecht and colleagues conducted a review of 321 articles, published between 2000 and 2019, to examine the evidence for precipitants of relapse in AUD.^91^ The review focused on 37 potential determinants of relapse in AUD, including gender, and identified the number of studies that found evidence for (or against) each relapse determinant. The review showed mixed results in terms of rates of relapse among men and women. Specifically, most studies (59%) included in the review found no gender differences in participants’ likelihood of relapse after treatment, but 41% of the studies did find gender differences and collectively suggested that women were less likely to relapse.^91^

In another review, Walitzer and Dearing indicated that rates of alcohol relapse did not differ among men and women, but evidence did indicate different predictors of relapse by gender.^92^ For women, being married, marital stress, interpersonal conflict, and negative affect were risk factors for alcohol relapse whereas for men, risk factors included isolation and both negative and positive affect. Being married was identified as a protective factor for alcohol relapse in men, and having more children in the home was protective for women. The gender difference in marital status in relation to alcohol relapse (protective for men, risk factor for women) is worth noting, given that women are more likely to be married to a spouse who drinks and men are more likely to be married to a light or non-drinker.^92^ Women also are more likely to drink to cope with marital conflict whereas men are more likely to report that their drinking contributes to marital conflict.^92^

### Various Forms of Recovery: Abstinence and Moderated Drinking

Gender differences in empirical studies on viability of non-abstinent forms of recovery have recently been studied. Analysis of gender differences in such studies needs to attend to different thresholds for risky or heavy drinking for men and women.^14^ Using Project MATCH data (30% female), four recovery profiles were generated at 3 years post-treatment: poor-functioning frequent heavy drinkers, poor-functioning infrequent heavy drinkers, high-functioning occasional heavy drinkers, and high-functioning infrequent non–heavy drinkers. No gender differences in profile assignment were found.^93^

In a study of three clinical trials for AUD—including data from Project MATCH, the COMBINE study, and the United Kingdom Alcohol Treatment Trial—several baseline variables were tested as predictors of low-risk drinking; gender was not found to be predictive.^94^ In a large epidemiological sample (41% female), gender differences in past-year likelihood of falling into one of six drinking patterns (ranging from abstinent recovery to five types of non-abstinent recovery) were examined. Women were more likely than men to be in the abstinent recovery or asymptomatic, low-risk drinking categories than in the persistent AUD category. Additionally, women were less likely than men to fall into the symptomatic, high-risk drinking category. These results persisted after adjustment for daily amount of alcohol used, severity of AUD, illicit drug use, SUD, and anxiety/depression.^95^

One study examined men and women with AUD between ages 55 and 77 in a private outpatient program.^96^ At 6-month follow-up, 79% of women reported abstinence from alcohol and drugs in the prior 30 days, compared to 54% of men. Among those not abstinent, no women reported heavy drinking in 30 days prior to follow-up, whereas non-abstinent men reported an average of 4 heavy-drinking days (a significant gender difference).

### Quality of Life During the Recovery Period

Issues such as co-occurring mental health conditions, social environment, sleep, and physical health are directly affected by problem drinking and are important independent outcomes reflecting quality of life (QoL). Literature reviews have shown that heavy drinking is associated with reduced QoL, which improves with reductions in drinking.^97^ There is some evidence that the association between drinking, recovery, and QoL may be moderated by sociodemographic constructs, including gender.^97^ Among women with AUD, both abstinence and moderate consumption of alcohol were associated with improved QoL over a mean follow-up of 46 months.^98^ Among 82 patients with AUD admitted for inpatient detoxification and assessed at baseline and 12 weeks later, women with AUD reported lower QoL (general health, psychosocial impairment) than men with AUD.^99^ These studies suggest that QoL be examined in gender differences to continue to address the relationship of QoL among women vis-à-vis reduction in drinking.

### Summary

Attention to gender differences among various forms of recovery (both in the 12-step model and in the treatment outcome literature)—including examination of abstinence, reduction of drinking, and/or secondary outcomes—has yielded some interesting results, but research is sparse so far. Predictors of relapse appear to differ between men and women, with women being more likely to relapse in response to interpersonal conflict and negative affect whereas men are more likely to relapse in response to isolation and both positive and negative affect. Also, although being married is a protective factor for men, it can act as a risk factor of relapse for women. Having at least one close friend to discuss drinking with is differentially helpful for women. Also, gender differences in treatment outcome and maintenance may depend on the outcome of interest (drinking or secondary outcomes) and the “form of recovery” studied.

## SEX AND GENDER DIFFERENCES IN AUD MECHANISMS OF BEHAVIOR CHANGE

There are several behavioral treatments now known to be efficacious for AUD, but there is almost no examination of gender differences in the AUD psychotherapy process and mechanisms of behavior change in this research literature. For example, the authors of this paper found 49 articles published between 2000 and 2012 (26 published since 2010) studying mechanisms of change in CBT, Motivational Interviewing, or MET or examining general therapeutic alliance as a mechanism of change. Of these 49 articles, 22 were review or non-empirical papers and did not mention gender. Of the 27 empirical studies, seven (26%) provided no sample breakdown by gender, one study (4%) had an all-female sample, and 17 (63%) had mixed-gender samples (albeit 11 of the 17 had samples that comprised at least two-thirds men). Furthermore, of these 17 mixed-gender studies, only five (29%) mentioned gender at all, typically as a statistical covariate. Since 2012, researchers have continued to examine mechanisms of change but generally have continued to ignore gender or used single-gender samples.

The Women’s Recovery Group (WRG), a treatment for women with SUD (including AUD), examined mechanisms of change between men and women. WRG was compared to a traditional mixed-gender Group Drug Counseling (GDC) treatment in Stage I^100^ and Stage II^101^ trials. The pilot study and RCT results indicated that WRG was at least comparable to a mixed-gender, traditional drug counseling group. Secondary analyses of the pilot study and/or RCT data tested affiliative (supportive, positive, or empathic) statements as WRG mechanisms of change. Women in WRG emitted more affiliative statements compared to both genders in the GDC condition. Affiliative statements were made more in WRG than GDC and were associated with better drinking outcomes during and 6 months after treatment for women, especially in WRG.^102^

Litt et al. studied Network Support Treatment (NST) for AUD, which is designed to help patients build social support networks for sobriety.^103^ Main treatment effects showed that men had a better treatment response than women. NST effects were mediated by changes in abstinence self-efficacy and number of abstinent friends for both men and women. Among those receiving NST, women reported less improvement in abstinence self-efficacy and fewer abstinent friends. Kelly and Hoeppner explored gender moderation of purported mediators, assessed at 9-month follow-up, of the effects of AA on drinking at 15-month follow-up among Project MATCH participants.^104^ Social self-efficacy and pro-abstainer social networks mediated AA’s effects on abstinence for both men and women, but a larger proportion of AA’s effect on treatment outcome was accounted for by these mediators for men (91%) than for women (57%). Additionally, although self-efficacy in positive social situations at 9-month follow-up was a mediator of the effect of AA on drinking at 15-month follow-up for men, it was not for women. Alternatively, self-efficacy not to drink in negative affect situations was a significant mediator for women, but not for men.

Recent studies have investigated potential mechanisms of behavior change among female-only samples receiving CBT for AUD (see McCrady, Epstein, and Folkus^6^ for review). For instance, using times-series network analysis to examine concurrent and sequential relationships among several putative mechanisms of change, Holzhauer et al. examined mechanisms of change in an RCT comparing a gender-neutral to a female-specific CBT for women with AUD.^105^ Higher self-confidence to abstain from drinking and increased use of alcohol-related coping skills were associated with less drinking in women in both CBT conditions. Women receiving female-specific CBT also reduced their drinking through decreased sociotropy (reactivity to others) and increased social support for abstinence. Changes in autonomy (importance of one’s independence and personal rights) were associated with higher self-confidence in abstinence, use of coping skills, and less drinking in both conditions, suggesting that increasing autonomy may be a treatment mechanism specifically for women.

Identifying mechanisms of behavior change in treatments for AUD is a critical research effort, as it provides an understanding of the active ingredients of effective treatments. Such an understanding provides clinicians information about the critical elements that should be provided for different populations and will aid dissemination of empirically based treatments.^106^,^107^ However, identifying such mechanisms has been difficult,^106^ and moderating factors, including sex and gender, may play an important role in how people change.

## DISCUSSION

Literature on gender and sex differences in AUD has grown exponentially since 1994. This has been particularly true regarding research on biopsychosocial risk and maintenance factors of AUD and treatment entry and gender-specific barriers to treatment for AUD. However, there is room for improvement regarding analysis and reporting of gender differences in treatment response for AUD and in mechanisms of drinking behavior change. Past reviews of gender differences in treatment outcomes have found mixed results and little evidence for systematic gender differences.^11^,^42^ However, many of the studies covered in these reviews were completed among patients in treatment for other substances or for alcohol and other substances, not AUD alone. Additionally, many of the studies reviewed were set in naturalistic settings rather than in randomized and/or controlled trials, and most studies simply did not recruit enough females and did not present data on gender differences even when there was a subset of female participants.

A recent review conducted by the RAND National Defense Research Institute examined 24 AUD RCTs to examine gender differences in outcome and found mixed results, with little evidence for systematic gender differences in treatment effects across studies.^108^ However, the authors of that review also stated: “Most notably, despite an extensive search and thorough screening procedure, we found very few studies reporting on gender differences, which hindered our analyses. . . . The review showed a profound lack of information on presence and absence of gender differences. We contacted authors and scrutinized numerous U.S. RCTs for differential effects for men and women but found very few relevant studies.”^108^ ^(p54)^

Our review and those by Greenfield and colleagues^42^ and Epstein and Menges^15^ all concur with this assessment—that there is not enough research on the topic of gender differences in treatment outcomes (psychotherapy or pharmacotherapy). There is not enough research on gender differences regarding the efficacy of specific treatments or enough research that examines secondary outcomes, aside from alcohol use, that are especially relevant to long-term recovery (e.g., co-occurring psychological disorders or symptoms, physical health, QoL, moderated drinking). Although some research suggests women may have better outcomes than men in recovery from AUD, multiple factors—including but not limited to sample size/percentage of women, severity of AUD, and motivation to change—may contribute to such findings and preclude conclusions at this point.

As suggested by Moyer and colleagues,^52^ future work would be enhanced by clearly delineated hypotheses about why gender differences might be expected in specific treatments—both in terms of treatment efficacy and in terms of mechanisms of behavior change. There has been substantial research on gender differences in risk and maintenance factors for AUD, and there is expanding research on female-specific treatment needs and approaches.^6^ The field of AUD treatment development may be well positioned to use this research on gender differences to propose hypotheses about and, perhaps more important, men and women might respond differentially to a given treatment. For example, Project MATCH formulated a priori gender matching hypotheses; although these were not confirmed in the direction expected, gender differences did emerge that were then available to inform continued research.

It is also important to note that even among the studies that examined sex and gender differences, the sample sizes of women were often small, and analyses were likely underpowered. Given the historical differences in prevalence of AUD among men and women, this may have been justifiable in the past. However, the convergence of prevalence rates for lifetime AUD among men and women no longer justifies such small samples of women in treatment. Although studies may recruit men and women, women often comprised less than 50% of the sample, which makes it difficult to examine gender differences. If gender is considered a moderating factor, there must be enough men and women to statistically power the examination of interaction effects. Thus, in conducting clinical trials it may be important to enroll comparable numbers of men and women, with sufficient power to properly examine gender differences.^108^ This includes using gender as a variable in randomization and examining gender-related co-occurring conditions and other secondary outcomes. The literature highlighted in this review provides substantial evidence that sex and gender differences impact the factors that are integral to AUD recovery—such as frequency and intensity of drinking, social functioning, physical health, risk for relapse, and possibly mechanisms of change—and therefore deserves to be considered in recovery research as the field moves forward.

Another consideration is single-gender treatment options, with female-only treatment most often a focus of research. This area of research has examined the delivery of treatment in a women-only setting, with or without including female-specific content (see McCrady et al.^6^ for a review). There is evidence for differential, positive outcomes for treatment delivered in women-only versus mixed-gender settings,^6^,^42^ but only when female-specific programming (i.e., content) also is provided. Thus, some argue that women-only treatment settings are not necessary, compared to mixed-gender settings, and at least one study of women in a residential treatment setting indicated that female-only treatment is not, at least initially, preferred by all female patients.^109^ However, consistent findings have suggested that women express satisfaction and preference for female-specific format and treatment content.^6^ Additionally, even if mixed-gender treatments were shown to be as good as or better than single-gender treatments, women-specific treatments are likely to enhance treatment access for many women.

## SUMMARY AND RECOMMENDATIONS FOR FUTURE RESEARCH

Gender differences in AUD treatment and recovery is an area in need of accelerated research. Specific areas of investigation are recommended:

- An overarching factor is the low engagement of men and women with AUD treatment. Gender differences may play important roles in understanding how, when, where, and why individuals seek care for AUD.
- Emerging research on digital and mobile technologies needs to include equal numbers of female and male participants and to analyze data by gender.
- Additional research is needed to test treatment access, retention, and outcomes for women versus men in primary care settings.
- Further research on gender-differentiated use of AA and other mutual help groups, and differences in treatment outcomes and mechanisms of change, is indicated.
- Rigorous, randomized trials for AUD on single-gender versus mixed-gender group settings with gender-specific programming are lacking.
- Another important contextual factor is a clarified definition of “recovery.” Variations in treatment goals and non-abstinent outcomes need to be examined, including gender as a moderating variable.
- Gender differences in secondary outcomes (such as co-occurring symptoms, interpersonal functioning, and quality of life) should be reported in AUD treatment outcome research.
- Research suggests gender differences in relapse precipitants. Furthering our understanding of biological, social, and psychological determinants of relapse based on gender has implications for personalized or tailored relapse prevention approaches.
- Clinical trials are mandated to recruit men and women, as well as analyze and report gender differences; however, the field needs to adhere more stringently to these mandates in future research. This involves consistent changes to methods such as intentional oversampling of women, randomization based on gender, and gender-specific analyses.

The research reviewed here provides ample reason to believe that men and women recover from AUD differently. It is important to test and report gender differences when studying mechanisms of change—mediators, moderators, and active therapeutic ingredients—in AUD treatments.
